# Biological Activity of Masked Endotoxin

**DOI:** 10.1038/srep44750

**Published:** 2017-03-20

**Authors:** Harald Schwarz, Jan Gornicec, Theresa Neuper, Maria Alejandra Parigiani, Michael Wallner, Albert Duschl, Jutta Horejs-Hoeck

**Affiliations:** 1 Department of Molecular Biology, University of Salzburg, Salzburg, Austria

## Abstract

Low endotoxin recovery (LER) is a recently discovered phenomenon describing the inability of limulus amebocyte lysate (LAL)-based assays to detect lipopolysaccharide (LPS) because of a “masking effect” caused by chelators or detergents commonly used in buffer formulations for medical products and recombinant proteins. This study investigates the masking capacities of different buffer formulations and whether masked endotoxin is biologically active. We show that both naturally occurring endotoxin as well as control standard endotoxin can be affected by LER. Furthermore, whereas masked endotoxin cannot be detected in Factor C based assays, it is still detectable in a cell-based TLR4-NF-κB-luciferase reporter gene assay. Moreover, in primary human monocytes, masked LPS induces the expression of pro-inflammatory cytokines and surface activation markers even at very low concentrations. We therefore conclude that masked LPS is a potent trigger of immune responses, which emphasizes the potential danger of masked LPS, as it may pose a health threat in pharmaceutical products or compromise experimental results.

Endotoxins are pyrogens originating from the outer layer of Gram-negative bacteria. Chemically, endotoxins are lipopolysaccharides (LPS), thus the terms “LPS” and “endotoxin” are often used interchangeably. However, LPS usually refers to a purified form of endotoxin which is used as a standard in industry and scientific research, whereas endotoxin is regarded as the naturally occurring complex of LPS present in the bacterial cell wall^1^.

The term “low endotoxin recovery” (LER) describes the inability of the limulus amebocyte lysate (LAL)-based assay to detect endotoxin due to a “masking effect”, which occurs as a result of formation of a macromolecular complex that prevents Factor C (the main component of the LAL coagulation cascade) from binding to endotoxin, even while positive controls show no evidence of test interference. LER-like phenomena were described as early as 1988 by Nakamura *et al*, who stated that “Triton X-100, which destroys LPS micelles, strongly inhibits the LPS-mediated activation of Factor C”^2^. However, the term “LER” was only recently introduced when Chen and Vinther reported on its problematic nature in 2013, whereupon the issue gained renewed attention from the scientific community^3^. The principal concerns are that undetected endotoxin contamination may pose a health threat or compromise scientific results. LER is also a topic of interest for regulators, including the US Food and Drug Administration (FDA), and several case studies were recently presented by Hughes *et al* (FDA) at the PDA Europe Conference Pharmaceutical Microbiology^4^. However, LAL assays, despite their limitations, currently remain the standard method (among others) for detecting and quantifying such contamination in recombinant proteins and other products used in medicine or science, as mandated by FDA and European Pharmacopeia regulations.

The onset of LER is dependent on factors like storage temperature, buffer formulations, endotoxin source (e.g. strain), and time^5^. As reported recently, LER can result from the use of chelating agents such as the commonly used sodium citrate or phosphate buffers in combination with detergents like polysorbate 20 (aka Tween-20) or octoxinol 9 (aka Triton X-100), or proteins such as bovine serum albumin (BSA)^5^. By removing divalent cations from LPS aggregates, chelators weaken the structure of these aggregates and enable detergents to intercalate, which leads to the formation of LPS monomers (Fig. 1)^5^.

The first step in the recognition of LPS involves its binding to LPS-binding protein (LBP), which can be found in the blood or extracellular fluid. LBP binds to LPS-rich parts of the bacterial membrane or to LPS aggregates (micelles) and thereby enables the extraction of LPS monomers by CD14, presumably by changing the arrangement of LPS aggregates^6^,^7^. CD14 then transports LPS to myeloid differentiation protein 2 (MD2) in the TLR4-MD2 complex^8^,^9^.

It was further demonstrated that the Sushi domains of Factor C, which are responsible for binding LPS, have a cooperative binding mechanism, meaning that more than one LPS molecule is required for Factor C activation^10^. Therefore, monomeric LPS may not be capable of sufficiently activating Factor C. Yet, the role of LBP and CD14 *in vivo* is to disaggregate LPS and deliver monomers to the TLR4-MD2 complex^11^. Based on the currently available information, it is not entirely clear whether monomers are biologically active *in vivo* or not. However, most studies agree that monomers fail to activate Factor C.

This discrepancy raised additional questions about study designs for investigation of LER. At several recent conferences, investigators claimed that endotoxin masking is exclusive to purified LPS, e.g. control standard endotoxin (CSE) or reference standard endotoxin (RSE), and does not occur when naturally occurring endotoxin (NOE) preparations are used^12^,^13^,^14^. The major argument is that CSE does not mimic a naturally contaminated product, because purified LPS does not exist in nature. LPS in native endotoxin is imbedded in cell wall fragments and may be protected from the dispersing effects of chelating buffers and surfactants and is therefore probably not prone to masking effects. Hence, it is argued that the observed masking effects would only represent an artefact emerging from faulty experimental design – as claimed in recent studies performed by Bolden *et al*.^14^,^15^. Since LPS contamination occurring in parenterals are NOEs and not CSEs, endotoxin masking should not be an issue in LAL testing.

However, LER continues to polarize the bio-industrial and scientific communities, thus propelling the need for extensive studies of this phenomenon and new methods to reliably detect masked endotoxin.

In the present study, we analysed the phenomenon of LER in a recombinant protein preparation produced in two different bacterial strains using various assays. We then reproduced LER by deliberately masking LPS. We analysed the effects of masked endotoxin of both CSEs and NOEs on human immune cells by measuring cytokine secretion and surface marker expression upon stimulation of primary human monocytes with masked and non-masked LPS.

## Results

The protein TTHA0849 (henceforth abbreviated as “Tth”) from the Gram-negative eubacteria *Thermus thermophilus* was recombinantly expressed in *E. coli* BL21 Star™ (DE3) and in ClearColi^®^ BL21 (DE3), the latter an *E. coli* strain with genetic deletions that result in LPS being modified to lipid IV_A_, which neither triggers an endotoxic response nor generates signals in the LAL or cell-based assays.

Both production batches of Tth were analysed in terms of their physicochemical properties. The proteins migrated as a single band in SDS-PAGE (Supplementary Figure S1a) and were monomeric in solution, as determined by dynamic light scattering (99.7% and 96.3%, respectively). The calculated monoisotopic mass of Tth is 16,942.92 Da, whereas the measured delta mass of BL21 Star™ (DE3) Tth was +0.7 Da and that of ClearColi^®^ Tth was +0.3 Da. Finally, circular dichroism (CD) spectroscopy produced identical curve shapes for the two batches, indicating comparable secondary structure content (Supplementary Figure S1b).

**Figure 2:**
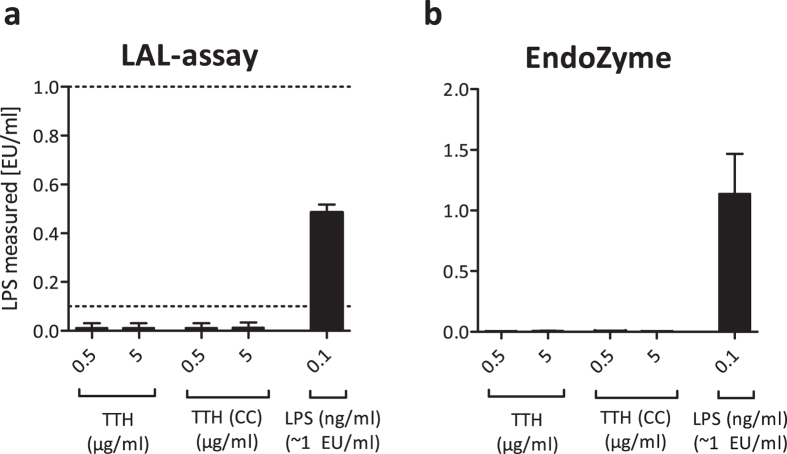
A recombinant protein (Tth) purified from *E. coli* or ClearColi (CC) shows no signs of LPS contamination in Factor C based assays. Two different concentrations of Tth purified from *E. coli* BL21 Star™ (DE3) or ClearColi^®^ (CC) BL21 (DE3) were analysed for LPS contamination using the LAL-assay (**a**) or the EndoZyme-assay (**b**). *E. coli* LPS 055:B5 (0.1 ng/ml or ~1 EU/ml) was used as a positive control. Detection range of the LAL-assay: 0.1 to 1 EU/ml, indicated by dotted lines. Detection range of the EndoZyme assay: 0.005 to 50 EU/ml. Mean and SD of three independent experiments are shown.

To test both recombinant protein preparations for endotoxin contamination, we utilized two assays: an endpoint chromogenic LAL assay and the EndoZyme recombinant Factor C assay. Two protein concentrations (0.5 and 5 μg/ml) were tested in both assays alongside an LPS control (0.1 ng/ml, which corresponds to ~1 EU/ml). As shown in Fig. 2, none of the tested protein concentrations showed indications of LPS contamination in either assay, whereas purified LPS was clearly detectable by both methods.

**Figure 3:**
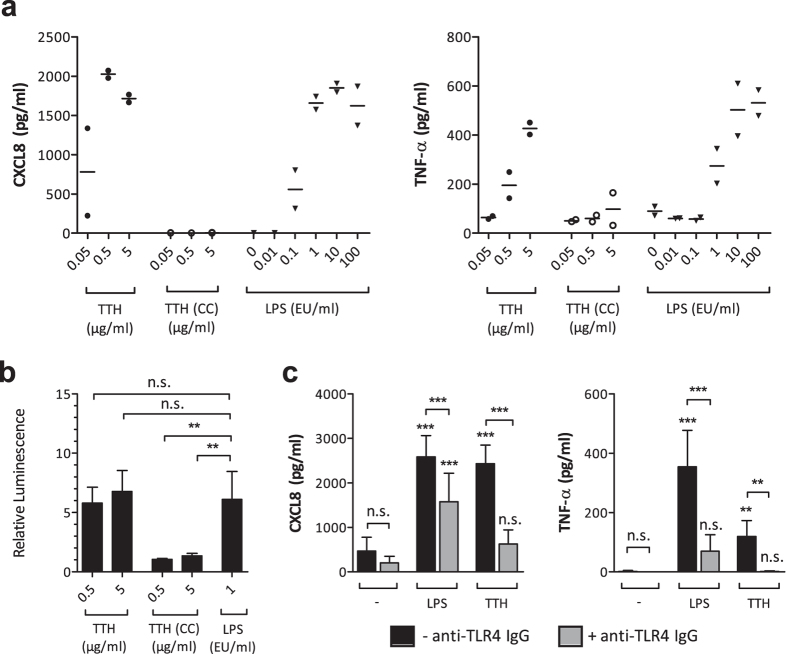
A recombinant protein (Tth) purified from *E. coli* induces pro-inflammatory mediators in monocytes and activates NF-κB in a TLR4-NF-κB-luciferase reporter gene assay. (**a**) Monocytes were stimulated with different concentrations of a recombinant protein (Tth) expressed in *E. coli* BL21 Star™ (DE3) or ClearColi^®^ (CC) BL21 (DE3), or with different amounts of LPS (0.1 ng/ml ~1 EU/ml). After 24 h of incubation, supernatants were analysed by ELISA. Single values of two independent experiments are shown. Mean values are indicated by bars. (**b**) HEK293 cells were transfected with an NF-κB-luciferase reporter plasmid and a plasmid-mix encoding a functional LPS receptor (TLR4, CD14 and MD2). 24 h after transfection, the medium was replaced and cells were stimulated as indicated (LPS: 0.1 ng/ml ~1 EU/ml). After another 24 h, luciferase activity was measured. Relative luminescence values were calculated relative to the untreated controls. Mean values and SD of at least 3 independent experiments are shown. (**c**) Monocytes were either left untreated (black bars) or treated with 1 μg/ml of a neutralizing IgG monoclonal antibody to human TLR4 for 1 h (gray bars). Thereafter, cells were either not further treated (−) or stimulated with 0.1 ng/ml (~1 EU/ml) LPS or 0.5 μg/ml Tth expressed in *E. coli* BL21 Star™ (DE3). After 24 h, supernatants were analysed by ELISA. Mean and SD of 7 independent experiments are shown. For statistical analysis, one-way ANOVA with a Tukey post-test was performed. **p < 0.01, ***p < 0.001, n.s. not significant.

In an effort to confirm that both protein preparations were largely endotoxin free, we stimulated human monocytes with increasing concentrations of Tth and compared the cytokine release to cells that had been activated with LPS. As shown in Fig. 3a, Tth produced in *E. coli* BL21 Star™ (DE3) elicited up-regulation of the pro-inflammatory mediators CXCL8 and TNF-α after 24 h of incubation, whereas Tth produced in ClearColi^®^ BL21 (DE3) showed no such effect. Because both proteins were expressed properly, the observed differences between the two proteins were most likely not caused by defective protein expression.

To further analyse the two protein batches for potential LPS contamination, we applied an NF-κB reporter gene assay, which is a sensitive method to measure very low amounts of LPS^16^. We transiently transfected HEK293 cells with plasmids encoding a functional LPS receptor (composed of TLR4, CD14 and MD-2) alongside an NF-κB-luciferase reporter plasmid. Twenty-four hours after transfection, two different concentrations of each Tth batch were added. After another 24 h, luciferase activity was measured. As shown in Fig. 3b, similar to LPS, Tth produced in *E. coli* BL21 Star™ (DE3) elicited a ~5-fold increase in TLR4-NF-κB-mediated luciferase activity compared to the untreated control. In contrast, Tth produced in ClearColi^®^ BL21 (DE3) showed no luciferase induction. As a control for non-TLR4-mediated NF-κB activation, cells were transfected with empty pcDNA plasmids instead of the LPS receptor plasmid mix, and this also resulted in no NF-κB activation (Supplementary Figure S2a).

To confirm that the observed cellular effects can be mainly attributed to LPS and the TLR4 signalling pathway, and are not caused by other contaminants (e.g. bacterial DNA), we performed TLR4 blocking experiments in monocytes. Monocytes were either left untreated or stimulated with 0.1 ng/ml LPS or 0.5 μg/ml Tth from *E. coli* BL21 Star™ (DE3), either alone or in the presence of a neutralizing anti-human TLR4 IgG-blocking antibody. After 24 hours, supernatants were harvested and again analysed for the expression of CXCL8 and TNF-α. Figure 3c shows that cytokine production induced by both LPS and Tth (produced in *E. coli* BL21 Star™) was significantly reduced upon addition of the TLR4-blocking antibody.

These results strongly indicated that, despite not being detectable by the two Factor C based assays above, Tth produced in *E. coli* BL21 Star™ (DE3) still contained endotoxin. During extraction of the recombinant proteins, a lysis buffer containing 25 mM sodium phosphate (pH 8), 0.1% Triton X-100, and 0.5 M Urea was used. After purification via chromatography, samples were stored in 10 mM sodium phosphate buffer. Both Triton X-100 and phosphate buffers have been shown to induce LER^5^; thus, the underlying mechanism contributing to NF-κB activation could be endotoxin masking.

Because the problematic nature of LER came to light only recently, the effects of masked LPS on the human immune system are currently not well studied. In a previous study, we showed that human immune cells, especially monocytes and CD1c^+^ blood dendritic cells, are highly LPS-sensitive and react to amounts as low as 20 pg/ml^16^. However, it is unclear whether or how strongly these cells react to masked endotoxin.

**Figure 4:**
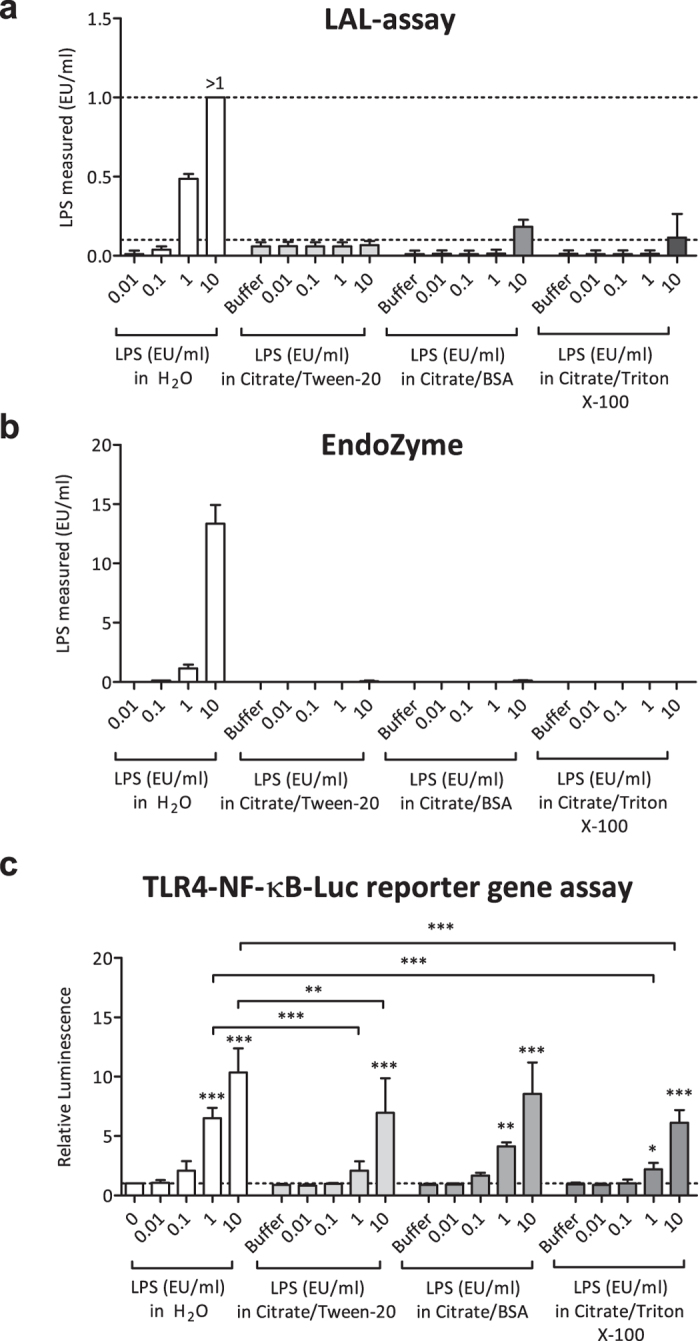
Detection of masked endotoxin. To investigate and reproduce the phenomenon of LER, three different masking-buffers (citrate/Tween-20, citrate/BSA and citrate/Triton X-100) were spiked with known amounts of LPS, and different LPS-detection assays were performed. LPS in H_2_O was used as a control. The LAL-assay (**a**), EndoZyme-assay (**b**) and TLR4-NF-κB-luciferase reporter gene assay (**c**) were performed. The reporter gene assay was conducted as described in Fig. 3, and cells were stimulated as indicated. Mean values and SD of three (**a,b**) or four (**c**) independent experiments are shown. For statistical analysis within each group, ANOVA with a Dunnett’s post-test was performed. For comparisons between different groups, ANOVA with a Tukey post-test was performed. p values shown represent the calculated difference to the corresponding concentration of LPS in H_2_O. *p ≤ 0.05; **p ≤ 0.01; ***p ≤ 0.001. The dotted lines indicate the range of the assays.

To analyse the potential masking effect of specific buffer components in more detail, we diluted known amounts of *E. coli* LPS 055:B5 in three different masking buffers containing a) 10 mM sodium citrate and 0.05% Tween-20, b) 10 mM sodium citrate and 10 mg/ml BSA, and c) 10 mM sodium citrate and 0.05% Triton X-100. We then analysed non-masked and masked LPS concentrations ranging from 0.01–10 EU/ml in the LAL-test, EndoZyme and TLR4-NF-κB-luciferase reporter gene assays. As shown in Fig. 4a, LPS in citrate/BSA and in citrate/Triton X-100 was only barely detectable in the LAL-test at a concentration of 10 EU/ml, which exceeds the range of the assay. LPS in citrate/Tween-20 was not detectable at all. Although the EndoZyme assay can detect a much larger range compared to the LAL-test, LPS was again not detectable in any of the three masking buffers (Fig. 4b). Only at the highest concentration used (10 EU/ml) did the EndoZyme assay detect LPS, but the detected values were between 0.5% and 5% of the applied concentration. Therefore, up to 99.5% of LPS in these buffers was not detectable, and presumably masked. Although this most likely resulted from LER due to endotoxin masking, another possible explanation for the observed effects could be that the masking buffers interfere with the assays and therefore simply inhibit the signals. To show that the observed effects were indeed LER and not assay interference, positive product controls (spiking of samples with defined amounts of LPS) were performed. Since endotoxin masking is a time-dependent effect^5^, addition of defined LPS spikes to the samples shortly before the measurement should result in measurable signals. Positive product controls (shown in Supplementary Figure S3) showed that the LPS spikes were indeed recovered; therefore, assay interference can be excluded.

To analyse whether masked LPS was still biologically active and able to activate cells via the LPS receptor, we applied the TLR4-NF-κB-luciferase reporter gene assay described above. As shown in Fig. 4c, LPS diluted in masking buffers, albeit hardly detectable by Factor C based assays, was still able to induce NF-κB activity. However, differences between LPS in water and LPS in masking buffers could be observed in this assay as well. At 1 EU/ml and 10 EU/ml, signals of LPS in citrate/Tween-20 and LPS in citrate/Triton X-100 were significantly lower than the signals obtained in the cells stimulated with the respective concentrations of LPS in water. However, the signals obtained from LPS in citrate/BSA were not significantly different compared to LPS in water. Again, controls utilizing empty pcDNA plasmids showed no signs of NF-κB activation (Supplementary Figure S2b).

**Figure 5:**
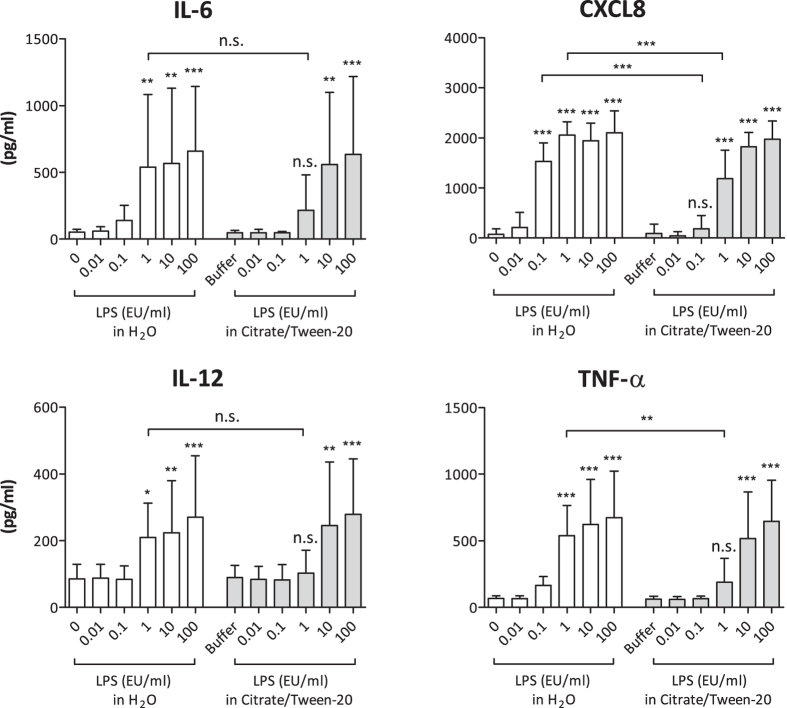
Cytokine expression of human monocytes stimulated with masked and non-masked LPS. 1 × 10^5^ monocytes were stimulated as indicated. After 24 h of incubation, supernatants were harvested and analysed via ELISA. Mean and SD of 8 independent experiments are shown. For statistical analysis within each group, ANOVA with a Dunnett’s post-test was performed. For comparisons between different groups, ANOVA with a Tukey post-test was performed. *p ≤ 0.05; **p ≤ 0.01; ***p ≤ 0.001; n.s. not significant.

In human monocytes and macrophages, exposure to LPS results in a rapid release of inflammatory cytokines and chemokines, including IL-6, IL-12, CXCL8 and TNF-α, which in turn attract and activate other immune cells, thus promoting the inflammatory cascade^17^. To test whether masked LPS activates primary human immune cells, we isolated human monocytes from buffy coats and stimulated them with different concentrations of masked and non-masked LPS. Because the masking buffer containing citrate and Tween-20 showed the highest masking capability in the LAL and EndoZyme assays (see Fig. 4), we used this masking buffer for all further experiments. Figure 5 shows that both non-masked and masked LPS are able to induce a concentration-dependent release of IL-6, CXCL8, IL-12 and TNF-α. Similar to the results obtained in the reporter gene assay, differences between LPS in water and LPS in citrate/Tween-20 were observed in this experiment as well. At a concentration of 1 EU/ml, LPS in citrate/Tween-20 induced significantly lower levels of CXCL8 and TNF-α. For CXCL8, this was also observed at a concentration of 0.1 EU/ml.

**Figure 6:**
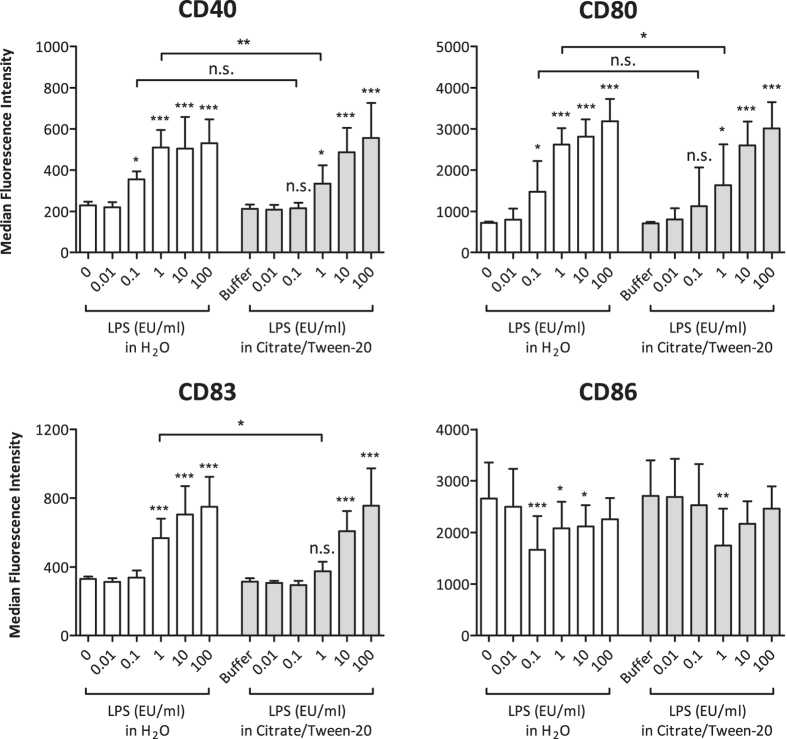
Flow cytometric analysis of surface activation markers on human monocytes stimulated with masked and non-masked LPS. 1 × 10^5^ monocytes were stimulated as indicated. After 24 h of incubation, cells were stained for the expression of CD40, CD80, CD83, and CD86 and subsequently analysed by flow cytometry. Median fluorescence intensity values of 1 × 10^4^ cells were recorded for each sample. Mean and SD of 5 independent experiments are shown. For statistical analysis within each group, ANOVA with a Dunnett’s post-test was performed. For comparisons between different groups, ANOVA with a Tukey post-test was performed. *p ≤ 0.05; **p ≤ 0.01; ***p ≤ 0.001; n.s. not significant.

Upon stimulation with LPS, monocytes up-regulate the activation markers and co-stimulatory molecules CD40, CD80 and CD83, whereas CD86 expression is down-regulated^18^,^19^. These co-stimulatory molecules are especially important during the activation of T cells^20^. To investigate whether stimulation with masked LPS could also produce an increase in surface activation markers on monocytes, we analysed the surface expression of CD40, CD80, CD83 and CD86 by flow cytometry. As shown in Fig. 6, both masked and non-masked LPS induced concentration-dependent up-regulation of CD40, CD80 and CD83, whereas CD86 expression was partially reduced. For CD40, CD80 and CD83, we observed a significant difference between masked and non-masked LPS at a concentration of 1 EU/ml. If we generalize the results shown in Figs 5 and 6, it seems that at a concentration of ~1 EU/ml, an approximately 10-fold higher amount of masked LPS is needed to induce similar effects as with non-masked LPS. Overall, our data show that masked LPS is a potent inducer of immune responses in primary human monocytes.

## Discussion

Endotoxins are complex organic molecules present in the outer membrane of Gram-negative bacteria. They are also potent immunostimulants in vertebrates. The minimal growth requirements of Gram-negative bacteria allow them to grow in clean water, saline, and buffers^1^, making them useful for biomedical and pharmaceutical applications. However, even commercially prepared proteins expressed by Gram-negative species may be contaminated by endotoxin up to several EU/ml^21^. Thus, especially in the fields of immunology and drug development, it is essential to be able to reliably detect endotoxin contamination. Besides the LAL-assay, another officially approved assay with increasing popularity is the monocyte activation assay (MAT). It was developed in 1995 by Wendel and Hartung and has several advantages as it is not restricted to LPS but also detects pyrogens from other sources such as Gram-positive bacteria^22^. Although the MAT circumvents the restriction of specificity for LPS by detecting immune-stimulating substances in general, the LAL assay remains the most sensitive and most economical test available^21^. Thus, every major pharmaceutical company uses it for quality control of their products.

Several recent studies have claimed that LER occurs only with purified LPS standards but not NOEs^12^,^13^,^14^. However, our results reported here support the view that LER occurs not only in CSEs, but also in NOEs. Our data demonstrate that both masked endotoxin contaminating our recombinant protein preparation (a NOE) as well as deliberately masked LPS (a CSE) are not detected in assays that rely on Factor C, as shown by the LAL-test and the EndoZyme assay. Moreover, our findings indicate that the effects of non-masked and masked LPS on human immune cells (monocytes in this study) are very similar, which demonstrates that endotoxin triggers immune responses whether it is masked or not. In some cases, however, masked endotoxin triggered lower responses than non-masked LPS.

A robust method for measuring very low amounts of LPS is the reporter gene assay utilizing HEK293 cells transiently transfected with a working LPS receptor and an NF-κB-luciferase reporter, as we described previously^16^. Our data show that this assay is partially able to detect masked endotoxin as well, and more accurately reflects the situation in primary human monocytes.

Interestingly, the detergent (in this case Triton X-100) was present only during extraction of the recombinant protein, not during storage in sodium phosphate buffer. This observation indicates that the masking effect is stable for long periods of time. This is also supported by the fact that the purified LPS stocks containing high LPS concentrations (ranging from 10 EU/ml to 100,000 EU/ml) that were deliberately masked using different masking buffers did not immediately lose the masking effect when diluted in water before the experiments were performed (a period of several hours). This shows that endotoxin masking is a stable effect that does not revert itself when the buffer formulation is changed. Rather, new methods to unmask endotoxin need to be developed to tackle this problem in the future.

In conclusion, LER is a serious concern in medicine and science, as Factor C based assays fail to detect masked endotoxin. Not only did we demonstrate that different buffer formulations lead to masking of LPS, but also that NOEs seem to be affected by LER and that masking is not limited to purified LPS standards. Masked LPS is biologically active and induces potent immune responses in both monocytes and an NF-κB-luciferase reporter gene assay. Therefore, Factor C based assays do not correlate with cell-based assays under LER conditions. Even when stimulated with low LPS concentrations (ranging from 0.01 EU/ml to 100 EU/ml), monocytes reacted by releasing vast amounts of cytokines and activating surface marker expression, which emphasizes the toxicity and potential danger of masked LPS. We therefore strongly recommend the use of cell-based assays when testing for masked LPS.

## Materials and Methods

All studies involving human cells were conducted in accordance with the guidelines of the World Medical Association’s Declaration of Helsinki. According to university guidelines, the project was approved by the head of the Department of Molecular Biology (Univ.-Prof. Dr. Hans Brandstetter), University of Salzburg, Austria.

### Expression of TTHA0849 (Tth) in *E. coli*

A codon-optimized variant of the gene coding for the protein TTHA0849 (Uniprot ID Q5SK03) from the extreme thermophile bacterial strain *Thermus thermophilus* HB8 was cloned into the expression vector pET30a^23^. The protein is a member of the StAR-related lipid-transfer (START) domain superfamily. The construct was transformed into competent *E. coli* BL21 Star™ (DE3) cells and protein expression was performed in LB medium supplemented with 2 mM MgSO4, 1% (v/v) glycerol, 0.2% (w/v) ammonium sulphate, 10 mM sodium phosphate pH 7.4 and 25 mg/L kanamycin. Cells were grown to an OD600 of 0.8 at 37 °C and thereafter induced with 0.5 mM isopropyl-β-D-thiogalactopyranoside (IPTG) for 18 h at 16 °C. After harvesting, cells were lysed in 25 mM sodium phosphate buffer pH 8, 0.1% Triton X-100, 0.5 M urea by three consecutive freeze/thaw cycles. Protein purification was performed using a phenyl sepharose column followed by a purification step with a DEAE sepharose column. Final polishing was performed using a Superdex 75 10/30 column (all GE Healthcare, Chicago, IL, US). Bacterial endotoxin was removed by consecutive wash steps of the protein with Triton X-114^24^. Purified proteins were stored at −20 °C in 10 mM sodium phosphate buffer pH 7.4.

Alternatively, the Tth construct was transformed into competent ClearColi^®^ BL21 (DE3) cells (Lucigen, BioCat GmbH, Heidelberg, Germany). Protein expression was performed in TB medium supplemented with 25 mg/L kanamycin. Cells were grown to an OD600 of 0.8 at 37 °C and thereafter induced with 0.4 mM IPTG for 18 h. Cells were harvested by centrifugation and lysed in 25 mM sodium phosphate buffer pH 8, 0.1% Triton X-100, 0.5 M urea by three consecutive freeze/thaw cycles. Protein purification was performed using a phenyl sepharose column followed by a DEAE sepharose column (all GE Healthcare). Purified proteins were stored at −20 °C in 10 mM sodium phosphate buffer pH 7.4.

### Physicochemical characterization of recombinant proteins

Protein purity was monitored by sodium dodecyl sulphate polyacrylamide gel electrophoresis (SDS-PAGE). Gels were stained with Coomassie Brilliant Blue G-250. Protein aggregation behaviour in solution was analysed by dynamic light scattering (DLS) using a DLS802 system from Viscotek (Viscotek Corp., Malvern Instruments, Malvern, Worcestershire, UK). The intact mass of recombinant proteins was determined by mass spectrometry using a Q Exactive Orbitrap mass spectrometer (Thermo Fisher Scientific, Waltham, MA, USA). For secondary structure analysis, CD spectra were recorded with a JASCO J-815 spectropolarimeter (Jasco, Tokyo, Japan) fitted with a PTC-423S/15 Peltier-type single-position cell holder at 20 °C.

### Masked LPS

For masking of LPS, defined amounts of gel-filtrated LPS from *E. coli* 055:B5 (Sigma-Aldrich Chemie, Steinheim, Germany) were spiked into three different buffer systems (see below) and stored for at least 7 days at 4 °C. Masking buffer 1: 10 mM sodium citrate (pH 7.5), 0.05% Tween-20 (Polysorbate-20; Sigma-Aldrich Chemie, Steinheim, Germany). Masking buffer 2: 10 mM sodium citrate (pH 7.5), 10 mg/ml BSA (Serva Electrophoresis GmbH, Heidelberg, Germany). Masking buffer 3: 10 mM sodium citrate (pH 7.5), 0.05% Triton X-100 (Sigma-Aldrich Chemie, Steinheim, Germany).

### Transient transfection and NF-κB-luciferase reporter gene assay

1.2 × 10^5^ HEK293 cells were seeded in 24-well plates in 500 μl DMEM (Sigma-Aldrich, Vienna, Austria) supplemented with 10% heat-inactivated (i.a.) foetal bovine serum (FBS; Merck-Millipore, Vienna, Austria), 2 mM MEM non-essential amino acids (NEAA; PAA, Pasching, Austria), 2 mM L-glutamine, 100 U/ml penicillin and 100 μg/ml streptomycin (all from Sigma-Aldrich, Vienna, Austria). After 24 h, transfection mixes were added to the cells. For that, 500 ng of DNA was diluted in Opti-MEM (Gibco, Thermo Fisher Scientific, Waltham, MA, USA), consisting of 100 ng LPS receptor mix (with a TLR4:MD2:CD14 ratio of 3:1:1) and 400 ng of NF-κB-luciferase reporter plasmid. As a control, empty plasmids were transfected instead of the LPS receptor mix, alongside the NF-κB-luciferase reporter plasmid (Figure S3). TLR4 (in pcDNA3), CD14 (in pcDNA3) and MD-2 (in pEF-BOS) were kind gifts from Andrei Medvedev and Douglas Golenbock. The NF-κB-luciferase reporter was kindly provided by Min Li-Weber and cloned into pGL3Neo in our laboratory. Additionally, Lipofectamine 2000 reagent (Invitrogen, Thermo Fisher Scientific, Waltham, MA, USA) was diluted in Opti-MEM according to the manufacturer’s instructions and added to the DNA mixes in a ratio of 1:1. 50 μl of the transfection mixes were then added to each well. After another 24 h, medium was replaced and cells were ready for stimulation. After 24 h of stimulation, cells were lysed and luciferase activity was measured in white 96-well flat-bottom microtiter plates using a Tecan Infinite 200 Pro microplate reader.

### Isolation of primary human monocytes

In this study, only immune cells derived from human buffy coats were used. Because our national regulations do not require informed consent in the case of anonymous blood cells discarded after plasmapheresis (buffy coats), no additional approval by the local ethics committee is required. Monocytes were isolated from buffy coats from healthy, anonymous donors (provided by the blood bank Salzburg, Austria) using the adherence method, as described^16^. Briefly, buffy coats were diluted in PBS and subjected to density gradient centrifugation using Histopaque-1077 (Sigma-Aldrich). After erythrocyte lysis and several washing steps, peripheral blood mononuclear cells (PBMCs) were seeded in 6-well plates at a density of 1 × 10^7^ cells per ml in monocyte medium (RPMI 1640 (Sigma-Aldrich, Vienna, Austria) supplemented with 10% i.a. FBS, 2 mM L-glutamine, 100 U/ml penicillin, 100 μg/ml streptomycin and 50 μM β-mercaptoethanol (Gibco, Thermo Fisher Scientific, Waltham, MA, USA) and incubated at 37 °C and 5% CO_2_ for 90 minutes. During this resting phase, monocytes adhere to the bottom of the wells. By extensive washing, the remaining non-adherent cells were removed. Cells were rested for 24 h at 37 °C and 5% CO_2_ and then re-plated at a concentration of 1 × 10^5^ cells per well in 24-well plates in 1 ml monocyte medium for further experimental procedures. The purity of monocytes was routinely analysed by flow cytometry.

### Flow cytometry

For flow cytometric analysis, cells were washed with ice-cold FACS buffer (PBS supplemented with 3% i.a. FBS) and stained in 50 μl FACS buffer containing appropriate amounts of fluorescent-labelled antibodies. As a control for nonspecific binding, one aliquot of cells was labelled with isotype-specific control antibodies in excess concentration. After 30 minutes of incubation and extensive washing, the median fluorescence intensity (MFI) of 1 × 10^4^ cells was recorded for every sample using a FACS Canto II flow cytometer and FACS Diva software (both from BD Biosciences, Vienna, Austria). Anti-human CD14-FITC (clone: MEM-15), mouse IgG1-FITC (PPV-06), mouse IgG2a-APC (PPV-04) and mouse IgG2b-PE (PLRV219) were purchased from ImmunoTools (Friesoythe, Germany). Anti-human CD40-APC (5C3), CD80-PE (L307.4), CD83-APC (HB15e) and CD86-PE (IT2.2) were acquired from BD Biosciences, Vienna, Austria. Mouse IgG1-APC isotype control (11711) was purchased from R&D Systems (Biomedica, Vienna, Austria).

### Enzyme-linked immunosorbent assay (ELISA)

Human IL-6, IL-8, IL-12 and TNF-α Standard TMB ELISA Development Kits were purchased from Peprotech, Vienna, Austria (cat. no. 900-T16, 900-T18, 900-T96, and 900-T25). Capture antibodies were coated on NUNC MaxiSorp flat-bottom 96-well plates (eBioscience, Affymetrix, Vienna, Austria) overnight at 4 °C. Blocking was performed for 1 h at room temperature (RT) using PBS supplemented with 1% BSA. Supernatants were added for 2 h at RT. Biotinylated detection antibody was added for 1 h at RT. Avidin-conjugated horseradish peroxidase was added for 30 min. Tetramethylbenzidine substrate (Sigma-Aldrich, Vienna, Austria) was added and the reaction was stopped by 2 M sulfuric acid. Colour intensity was measured at 450 nm and a reference wavelength of 650 nm was subtracted. The respective standards were used to calculate total protein concentrations.

### EndoZyme assay

The EndoZyme recombinant Factor C (rFC) assay (generously provided by Hyglos GmbH, Bernried am Starnberger See, Germany) was used according to the manufacturer’s instructions. Briefly, standards or samples (including controls) were pipetted into Nunclon 96 Flat Bottom Black Polystyrol LumiNunc Fluoronunc plates. After that, the reaction mix, containing the substrate and the enzyme (rFC), was added and mixed. Fluorescence was measured using a Tecan Infinite 200 Pro microplate reader which was pre-warmed to 37 °C, using the following settings: Excitation filter (nm/band): 380/9, emission filter: 440/20, 10 readings per well, gain: 75. After the first measurement, the plate was incubated for 90 minutes at 37 °C, followed by a second measurement (using the same settings as before). LPS-concentrations were determined by calculating a standard curve using a 4-parameter logistic non-linear regression model utilizing the formula y = (A − D)/[1 + (X/C)^B^] + D. To control for test interference of buffer components, spike controls were performed (Figure S3b). For that, buffer samples were spiked to a final concentration of 5 EU/ml with LPS. A result was considered valid if the spike recovery was in the range of 50–200%.

### LAL assay

The Endpoint Chromogenic LAL assay (Lonza Group Ltd, Szabo Scandic, Vienna, Austria) was used according to the manufacturer’s instructions. Briefly, 50 μl of LAL-enzyme was added to 50 μl of standards or samples in 96-well flat-bottom plates and carefully mixed. After 10 minutes of incubation at 37 °C, 100 μl of pre-warmed substrate was added. The plates were then incubated for 6 more minutes at 37 °C. To stop the reaction, 100 μl of 25% acetic acid was added to each well. Absorption was measured at 405 nm using a Tecan Infinite 200 Pro microplate reader. LPS concentrations were calculated from the standards using a linear regression model: y = A*x + B. In the experiments shown in Figs 2a and 4a, the accompanying standard “*E. coli* 0111:B4 Endotoxin” was used to calculate the regression model, whereas in Supplementary Figure S3a, “*E. coli* LPS 055:B5” (Sigma-Aldrich) was used. Again, spike controls were performed to control for test interference (Figure S3a). The result was considered valid if the difference between these two endotoxin values equalled the known concentration of the spike ± 25%.

### TLR4 blocking experiments

For TLR4 blocking experiments, 1 × 10^5^ human monocytes per well were plated in 24-well plates in 1 ml of medium and either left untreated or incubated with 1 μg/ml of a neutralizing IgG monoclonal antibody to human TLR4 (Invivogen, San Diego, CA, USA; Cat# mabg-htlr4) for 1 hour at 37 °C. Thereafter, the respective concentrations of *E. coli* LPS 055:B5 (Sigma-Aldrich) or Tth (purified from *E. coli* BL21 Star™ (DE3) were added. After another 24 h, supernatants were harvested and analysed by ELISA.

### Statistical analysis

Statistical analyses were performed using GraphPad Prism Software Version 5.01 (GraphPad Software Inc., La Jolla, CA, USA). If not stated otherwise, for the analysis within each group, ANOVA with a Dunnett’s post-test was performed (compares each value to the respective control value; e.g. the buffer). These statistics are indicated as stars directly above the bars. For comparisons between different groups (e.g. 1 EU/ml LPS in water vs. 1 EU/ml LPS in citrate/Tween-20), ANOVA with a Tukey post-test was performed. p values of less than 0.05 were considered statistically significant.

## Additional Information

**How to cite this article**: Schwarz, H. *et al*. Biological Activity of Masked Endotoxin. *Sci. Rep.* **7**, 44750; doi: 10.1038/srep44750 (2017).

**Publisher's note:** Springer Nature remains neutral with regard to jurisdictional claims in published maps and institutional affiliations.

## Supplementary Material

Supplementary Information

## Figures and Tables

**Figure 1:**
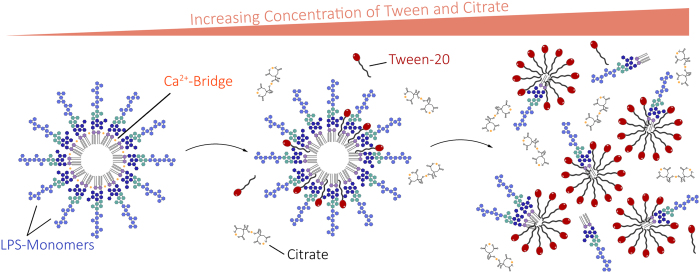
Schematic process of LPS masking. In aqueous solution, LPS naturally forms aggregates such as micelles or vesicles. Addition of chelators (such as citrate) weakens the aggregate structure by removing divalent cations. Detergents like polysorbate (e.g. Tween-20) then intercalate into the aggregates. Eventually, the aggregates completely disperse into monomers. Factor C, which triggers the LAL-coagulation-cascade, cannot be activated by LPS-monomers, leading to false negatives when quantifying masked LPS^5^.
