# Supplementary material for: Biological Activity of Masked Endotoxin
Source: Sci Rep. 2017 Mar 20;7:44750. doi: 10.1038/srep44750 (PMC5357793; doi:10.1038/srep44750)
Supplement: Supplementary Information [file srep44750-s1.doc]

**Supplementary Information**

**Biological Activity of Masked Endotoxin**

Harald Schwarz^1^, Jan Gornicec^1^, Theresa Neuper^1^, Maria Alejandra Parigiani^1^, Michael Wallner^1^, Albert Duschl^1^ and Jutta Horejs-Hoeck^1^*

^1^ Department of Molecular Biology, University of Salzburg, Salzburg, Austria

*Corresponding author:

Jutta Horejs-Hoeck

Department of Molecular Biology

University of Salzburg

Hellbrunnerstr. 34

A-5020 Salzburg

Austria

Tel: +43 (0)662 / 8044-5736

Fax: +43 (0)662 / 8044-5751

E-mail: jutta.horejs-hoeck@sbg.ac.at

orcid.org/0000-0002-0984-204X

**Supplementary figures**

**Supplementary figure S1**


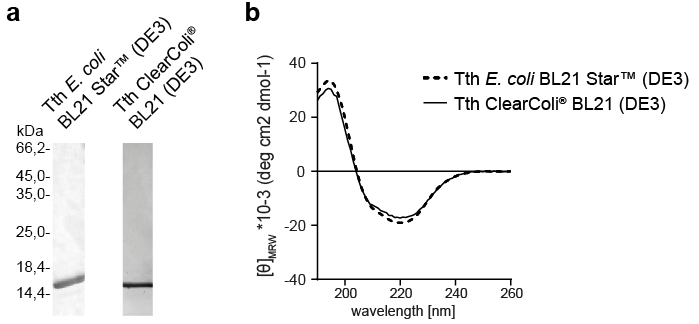


**Figure S1: Physicochemical characterization of Tth production batches.**

SDS-PAGE analysis of recombinant Tth batches (a). CD spectra were recorded at 20°C and are presented as mean residue molar ellipticity after baseline correction (b).

**Supplementary figure S2**

**Figure S2: NF-κB-luciferase reporter gene assay empty vector controls.** As a control for TLR4-independent NF-κB activation, HEK293 cells were transfected with 100 ng empty pcDNA3 plasmid alongside the NF-κB-luciferase reporter plasmid. Cells were then stimulated as indicated. Empty vector controls for the experiments performed in Figure 3b (a) and Figure 4c (b) are shown.

**Supplementary figure S3**

**Figure S3: Positive product controls (spiking).** To exclude assay interference of buffer components, spiking was performed for selected samples. For the LAL-test (a), buffers were used as in Figure 4, or spiked with 0.4 EU/ml LPS shortly before performing the assay. The known concentration of the spike should equal the difference between the two measured values ±25%, indicated by whiskers. For the EndoZyme assay (b), spiking controls were again performed by spiking the buffer samples to a final concentration of 5 EU/ml with LPS. Test interference can be excluded when the spiked samples stay within the range of 50 – 200% of the spike concentrations, indicated by the dotted lines.
